# Clinical features of patients with previous spontaneous rupture of ovarian endometrioma operated electively: a case-control study

**DOI:** 10.1186/s12978-023-01702-z

**Published:** 2023-10-21

**Authors:** Zhiyue Gu, Xiaoyan Li, Yi Dai, Jinghua Shi, Yushi Wu, Chenyu Zhang, Qiutong Li, Hailan Yan, Jinhua Leng

**Affiliations:** 1 Department of Obstetrics and Gynecology, Peking Union Medical College Hospital, Chinese Academy of Medical Sciences and Peking Union Medical College, No. 1 Shuaifuyuan, Dongcheng District, Beijing, 100730 China; 2 National Clinical Research Center for Obstetric & Gynecologic Diseases, Beijing, China

**Keywords:** Endometriosis, Ovarian endometrioma, Acute abdominal pain, Rupture, Elective surgery

## Abstract

**Background:**

The aim of the study is to investigate the proportion and clinical features of previous spontaneously ruptured ovarian endometrioma among women who underwent elective surgery for endometrioma.

**Methods:**

This retrospective study was based on a cohort of elective surgeries for endometrioma performed by the same gynecologic team at Peking Union Medical College Hospital from January 2017 to October 2022. Patients diagnosed with previous spontaneously ruptured endometrioma during elective surgery were enrolled in the ruptured group. In the same cohort, patients with unruptured endometrioma treated during the same period were selected as the unruptured group by 1:2 matching according to age. Demographic and clinical information were collected and compared between two groups.

**Results:**

A total of 422 patients in the cohort were diagnosed with endometrioma. There were 38 patients (9.0%) in ruptured group and 76 patients in unruptured group. All enrolled participants were treated by laparoscopic surgery. In ruptured group, 86.8% patients had a history of acute abdominal pain, which was only 13.2% in unruptured group (*P* < 0.001). Compared to unruptured group, patients diagnosed with ruptured endometrioma had a lower BMI (*P* = 0.021), larger maximum diameter of endometrioma (*P* = 0.040), higher proportion of cul-de-sac partial obliteration rather than complete obliteration (*P* = 0.003).

**Conclusions:**

Spontaneous rupture of endometrioma is not rare. The proportion of spontaneous rupture of endometrioma in our study was higher than that reported in the literatures. In women with endometrioma, the onset of acute abdominal pain should be considered a rupture of cyst, especially in patients with big cysts.

## Background

Endometriosis (EM) is defined as the growth of endometrium outside the uterine cavity [1], which often occurs in the pelvic cavity and can be divided into three categories: superficial peritoneal endometriosis (PEM), deep endometriosis (DE), and ovarian endometriosis (ovarian endometrioma, OMA) [2], and there are often many types of endometriosis at the same time. As a common gynecological disease, EM debilitates about 10% reproductive women [1], and is observed in 50–80% of women with pelvic pain and up to 50% of women with infertility [3]. OMA accounts for 17–44% of all kinds of EM cases [4], which usually forms cysts that enlarge with the increase of intracystic pressure and proliferation of the ectopic endometrium within the cyst wall [5]. There were various hypotheses about the development of endometrioma. Deprest et al. confirm that, in most cases, the endometrioma was formed by invagination of the cortex and that active endometrium implants were located at the site of invagination. Therefore, endometriomas are defined as pseudocysts which are formed by an extraovarian hematoma, surrounded by duplicated ovarian parenchyma [6]. Donnez et al. considered that, unlike PEM, which is likely the consequence of the implantation of shed endometrium, ovarian endometriotic cysts could arise from invagination and metaplasia of ovarian epithelium lining [7]. Some experts also thought that the ovulation is crucial in the development of endometriotic cysts. It has been proposed that during menstruation, an enlarged corpus luteum rupture may allow the retrograde menstrual endometrium to enter the ruptured ovarian tissue. The residual endometrial tissue is nourished by the corpus luteum cells after the wound closed to develop into a blood-containing endometrioma [8].

OMA is usually thin-walled, and its spontaneous rupture or partial leakage of chocolate-colored contents from its wall is not uncommon and may recur [5]. Spontaneous rupture of OMA can occur during menstruation, late menstrual cycle, ovulation period [5], and even during pregnancy [9]. Patients usually develop acute abdominal pain, sometimes combined with nausea and vomiting, sweating, and fever, which usually last from a few minutes to a few hours. Emergency surgery may be an option for patients with severe symptoms or a large amount of fluid leakage in the pelvic cavity, as indicated by ultrasound. However, there is still a proportion of people who choose conservative treatment during the onset of acute abdominal pain, and the subsequent elective operation shows signs of ruptured OMA.

In our cohort of OMA treated by elective surgery, some patients were diagnosed with spontaneously ruptured OMA. The aim of the present study was to investigate the prevalence and clinical features of spontaneously ruptured OMA among women who underwent elective surgery for OMA and compare them with those without ruptured OMA.

## Materials and methods

### Participants

**Fig. 1:**
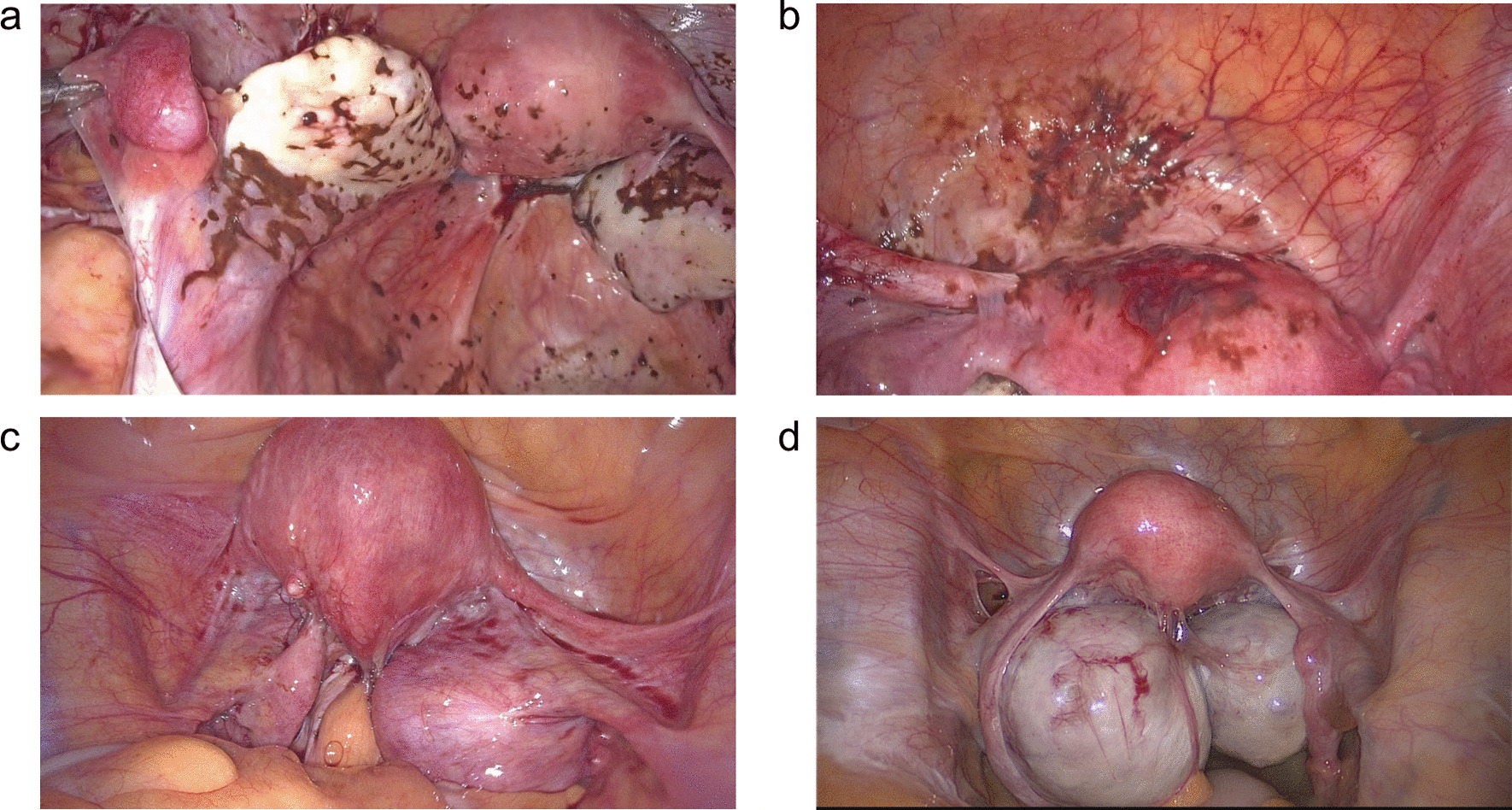
**a, b** Densely distributed chocolate-like stainings on the surface of the pelvic peritoneum and pelvic organ surfaces in ruptured group. **c**, **d** Laparoscopic view of control group

The aim of this study is to investigate the proportion and clinical features of previous spontaneously ruptured OMA among women who underwent elective surgery for OMA. This retrospective study was conducted on a derivation cohort of patients with OMA who underwent elective surgery at Peking Union Medical College Hospital (PUMCH) from January 2017 to October 2022. All the diagnosis of OMA were verified by histopathology, and the cases combined with borderline ovarian tumors and/or reproductive malignant tumors were excluded. During operation, the diagnosis of spontaneous rupture of OMA is based on careful inspection of pelvic and abdominal cavities. The signs of rupture included: densely distributed chocolate-like stainings on the surface of the peritoneum (peritoneum of fossa vesicouterina, posterior pelvic peritoneum, cul-de-sac, lateral pelvic peritoneum), omentum, or pelvic organ surfaces (Fig. 1a, b). In the same cohort, unruptured patients treated by elective surgery during the same period were chosen as the control group by matching them 1:2 according to age (Fig. 1c, d). The study was approved by the Ethics Committee of PUMCH (I-22PJ661), and all included patients provided their consent.

### Data collection

The retrospective data of patients in the study mainly consisted of preoperative and intraoperative information. Preoperative information of patients included age, body mass index (BMI), gravity, parity, cesarean section history, initial or recurrent OMA surgery, preoperative infertility, dysmenorrhea (Visual Analog Score, VAS), acute abdominal pain history (number, onset time, duration, accompanying symptoms and treatment), preoperative CA125, with or without CA19-9 levels, the maximum diameter of a single OMA by ultrasound, and preoperative medical treatment. Intraoperative information included the size of unilateral or bilateral cyst, cul-de-sac obliteration, co-existing PEM, DE, adenomyosis or uterine fibroids, EM scoring and staging according to the American Society for Reproductive Medicine revised staging system (r-ASRM), as well as surgery time and blood loss.

### Statistical methods

Statistical analysis was performed using SPSS Version 22.0 (SPSS Inc., Chicago, IL, USA). Continuous variables were expressed as mean ± standard deviation (M ± SD) or median with interquartile range (IQR), and categorical variables were showed as percentage. Quantitative variables were compared using the *t* test or Mann-Whitney *U* test, and qualitative variables were compared by the chi-square or Fisher’s exact test. A two-tailed *P*-values < 0.05 was considered statistical significance. We didn’t deal with missing data.

## Results

### Demographic and clinical information of the patients with spontaneously ruptured OMA

**Fig. 2:**
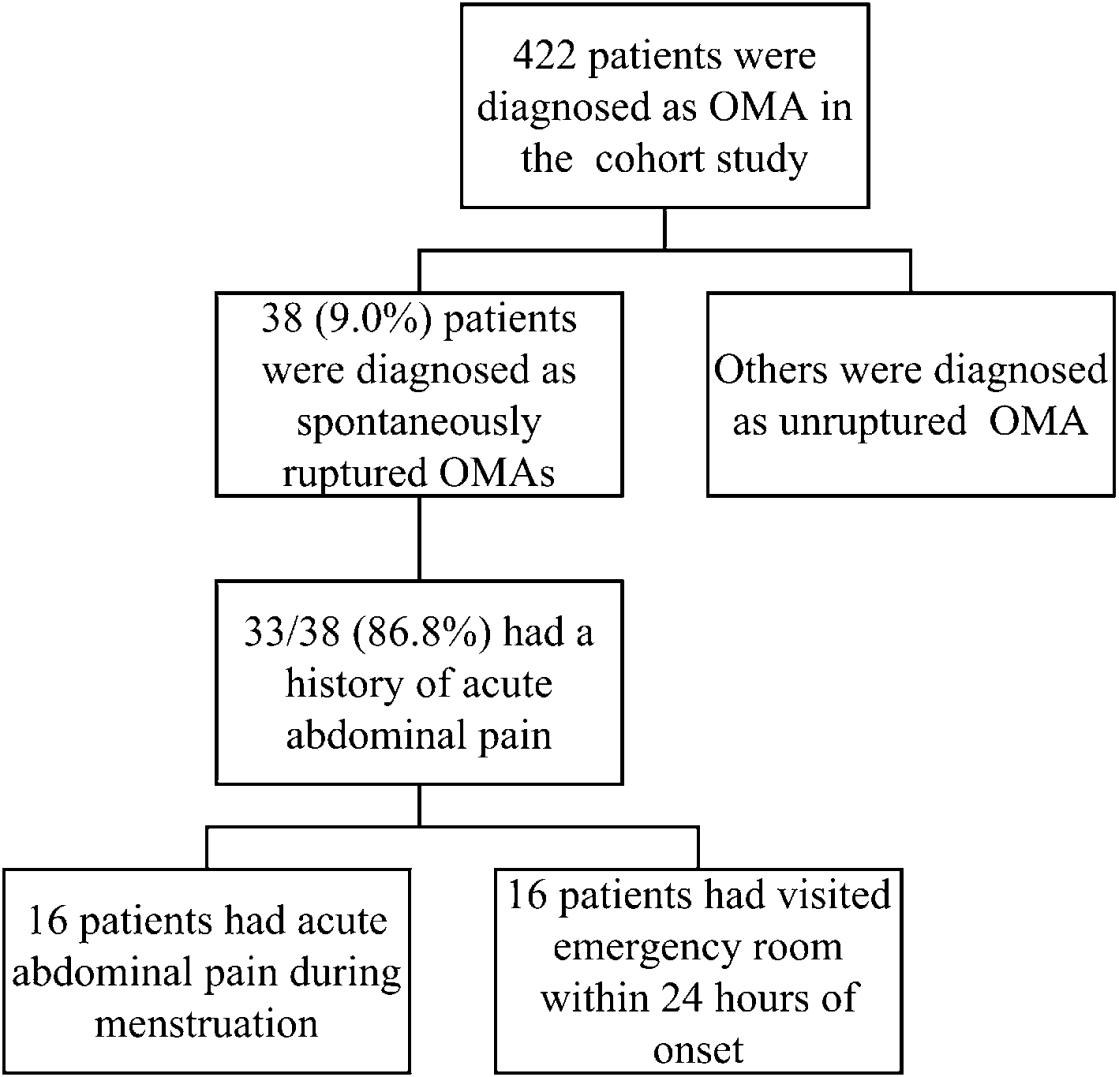
The flow diagram of included patients

A total of 422 patients in the cohort of elective surgery were included, 38 (9.0%) patients were diagnosed as having spontaneously ruptured OMAs. 33 (86.8%) patients had a history of acute abdominal pain (AAP) and 10 (30.3%) patients had experienced AAP more than once. The abdominal pain usually has no obvious trigger, and only 2 patients had AAP after exercise. 27 (81.8%) patients had AAP within 1 year, and 20 (60.6%) patients had it within 6 months. 16 patients had gone to the emergency room within 24 h at the onset of AAP. The flow diagram of included patients is shown in Fig. 2.

The symptoms of AAP onset varied among patients. For patients with a history of multiple AAP episodes, the symptoms at each onset might also be different. Some patients might suffer severe abdominal pain within 1 min, and sometimes the pain gradually aggravates to reach its peak in few minutes. The duration of the pain ranged from 5 to 10 min to a few hours. Patients with longer pain duration (1 day to 1 week) might have persistent mild to moderate pain after severe abdominal pain. Some patients have combined fever, nausea, vomiting, anal distension, and diarrhea during the onset of AAP and even been suspected of having digestive diseases. With ectopic pregnancy excluded, the diagnosis mainly consisted of a possible rupture of OMA, a possible rupture of the luteal corpus, pelvic inflammatory disease, and appendicitis. For patients with a known history of OMA, attending doctors were more likely to diagnose possible rupture of OMA and made preoperative preparations in advance. Not all the patients in the ruptured group had fever at the onset of AAP, only 10 had a definite history of fever, 9 of whom had a temperature of 37.9 ± 0.6 (37.3–39.0) °C. All patients visiting the emergency room for AAP resolved spontaneously or responded to conservative management. In addition, more than half of patients were relieved spontaneously, with or without painkiller, rather than visiting doctors. The AAP details of 33 patients in the ruptured group are shown in Table 1.

**Table 1:** Clinical features of acute abdominal pain of 33 patients diagnosed with spontaneous rupture of OMA

Characteristics	Number of patients	%
Total	33	
AAP as initial symptom for OMA, n (%)	9	27.3
AAP onset > 1, n (%)	10	30.3
Develop in menstrual period, n (%)	16	48.5
Visiting emergency room within in 24 h	16	48.5
Course of last AAP to surgery (mth)		
Median (IQR)	5.0 (3.0– 11.5)	
≤ 3	13	39.4
3– 6	7	21.2
6–12	7	21.2
> 12	6	18.2

AAP, Acute abdominal pain; OMA, Ovarian endometrioma; IQR, Interquartile range

### The comparison between spontaneously ruptured and unruptured OMA group

In the same cohort, 78 unruptured patients treated during the same period were selected as the unruptured group by 1:2 matching according to age. Obviously, the unruptured group has a lower percent of AAP history, which was only 13.2% (*P* < 0.0001). The ruptured group had a lower BMI of 20.4 ± 3.0 compared to 21.8 ± 3.0 in the unruptured group (*P* = 0.021). The features of pelvic pain, including dysmenorrhea degree and the prevalence of chronic pelvic pain had no significant difference between the two groups. Whether unilateral or bilateral OMAs, if we choose the maximum diameter of OMA by ultrasound as a representative, and we found that the average of the maximum diameter of OMA in the ruptured group was larger than that in the unruptured group (7.3 ± 1.7 vs. 6.6 ± 1.9, *P* = 0.04). Although the median of CA125 values were higher in the ruptured group, the results were not statistically different. In terms of the intraoperative information, there were no significant differences in the proportion of bilateral OMA, co-existing with PEM, DE, adenomyosis, and uterine fibroid, as well as surgery time and blood loss between ruptured and unruptured groups. In our study, the r-ASRM stage of all patients belonged to III or IV stage and there was no difference between the two groups, but the cul-de-sac of the ruptured group was more likely to be in partial obliteration rather than in complete obliteration (*P* = 0.007). The r-ASRM score in the unruptured group was higher than that in the ruptured group, but there was no statistical difference (52.6 ± 25.8 vs. 44.6 ± 22.5, *P* = 0.089). The results of the comparison between the two groups are shown in Table 2.

**Table 2:** Comparison of variables between spontaneous ruptured OMA and unruptured groups

Variables	Ruptured group(n = 38)	Unruptured group(n = 76)	*P*
Age (y), (M ± SD, range)	31.6 ± 5.1 (23–46)	31.7 ± 5.1 (23–46)	0.937
BMI (kg/m^2^), (M ± SD, range)	20.4 ± 3.0 (16.0–27.7)	21.8 ± 3.0 (16.7–31.2)	0.021*
Parity ≥ 1, n (%)	8 (21.1)	20 (26.3)	0.538
CS history, n (%)	4 (10.5)	14 (18.4)	0.276
Recurrence OMA surgery, n (%)	1 (2.6)	2 (2.6)	1.000
Infertility, n (%)	7 (18.4)	12 (15.8)	0.771
Primary infertility	5	10	
Second infertility	2	2	
Chronic pelvic pain, n (%)	5 (13.2)	6 (7.9)	0.907
Dysmenorrhea			0.300
None	9 (23.7)	14 (18.4)	
Mild (VAS 1–3)	6 (15.8)	12 (15.8)	
Moderate (VAS 4–6)	13 (34.2)	17 (22.4)	
Severe (VAS 7–10)	10 (26.3)	33 (43.4)	
OMA duration (mth), median (IQR)	21.0 (5.5–36.0)	12.0 (5.0–36.0)	0.680
AAP history, n (%)	33 (86.8)	10 (13.2)	< 0.001*
CA125 (IU/ml), n	37	70	
Median (IQR)	72.6 (37.8–154.5)	61.2 (34.9–113.9)	0.286
CA199 (IU/ml), n	34	63	
Median (IQR)	36.8 (14.7–74.7)	25.5(17.0–38.7)	0.176
Preoperative GnRH-a, n (%)	16 (42.1)	31 (40.8)	0.937
Max diameter of OMA by US (cm)	7.3 ± 1.7	6.6 ± 1.9	0.040*
Left cyst in surgery, n (%)	28 (73.7)	65 (85.5)	0.124
Right cyst in surgery, n (%)	30 (79.0)	53 (69.7)	0.297
Bilateral cyst in surgery, n (%)	20 (52.6)	42 (52.3)	0.790
Endometrial polyp, n (%)	2 (5.3)	9 (11.8)	0.432
Peritoneal EM, n (%)	8 (20.1)	23 (30.3)	0.297
Deep EM, n (%)	16 (42.1)	33 (43.4)	0.894
Uterine fibroid, n (%)	8 (21.1)	20 (26.3)	0.538
Adenomyosis, n (%)	6 (15.8)	18 (23.7)	0.330
Cul-de-sac obliteration			0.007*
Partial	22 (57.9)	21 (27.6)	
Complete	10 (26.3)	37 (48.7)	
r-ASRM score, (M ± SD, range)	44.6 ± 22.5 (20–96)	52.6 ± 25.8 (20–112)	0.089
r-ASRM stage, n (%)			0.274
III	17 (44.7)	26 (34.2)	
IV	21 (55.3)	50 (65.8)	
Surgery time (min), median (IQR)	60 (60–90)	70 (60 –90)	0.303
Blood loss (ml), median (IQR)	50 (30–50)	30 (30–50)	0.750

M ± SD, Mean and standard deviation; BMI, Body mass index; CS: Cesarean section; OMA: Ovarian endometrioma; VAS: Visual analog scale; IQR, Interquartile range; AAP: Acute abdominal pain; GnRH-a, Gonadotropin-releasing hormone agonist; US: Ultrasound; EM, endometriosis; r-ASRM, American Society for Reproductive Medicine revised staging system

## Discussion

In this cohort study of patients with OMA who underwent elective surgery at PUMCH, some were diagnosed with spontaneous rupture history during surgery. The proportion of spontaneous rupture of OMA in our study was almost one out of ten women, suggesting that spontaneous rupture of OMA is not rare.

In a multicentral and prospective study, the cumulative incidence of cyst rupture within 2 years of follow-up was 0.2% among 1919 patients with a new adnexal mess at recruitment [10]. As a very common gynecological disease and because of the formation of adhesions, OMA is not prone to twisting but has a chance of rupture. In previous studies, spontaneous rupture of OMA was also regarded as a rare condition, and the prevalence was reported to vary from 2.6 to 4.2% in the literatures [9, 11, 12]. Otherwise, the prevalence of spontaneous rupture of OMA in our study (9.0%) was higher than previous reports, which were mostly based on emergency surgeries and might underestimate the prevalence of this condition. All our included patients were all treated through elective surgeries.

In our study, patients in the ruptured group seemed to have a lower BMI (20.4 ± 3.0 compared to 21.8 ± 3.0, *P* = 0.021), the mean BMI was similar to the result of Huang et al’s study, which was 20.01 ± 2.65 [13]. According to our study, spontaneous rupture of OMA probably most likely occurred during menstruation (Table 1). There were nearly half the patients who had a history of acute lower abdominal pain during menstruation (16/33). This result might be a little different from the result of Pratt et al. [5], in which there was only 7/37 patients suffering the onset of acute abdominal distress during the 1–5 days of the menstrual cycle.

By comparing the two groups, patients with spontaneous ruptured OMA had a larger mean maximum diameter of OMA than the unruptured group. Because of retrospective studies, there is no extinct conclusion about the diameter of OMA when it is prone to rupture. A retrospective study of 13 patients with spontaneously ruptured OMA confirmed by surgery showed that CT examination before operation revealed that the average maximum diameter of the cyst was 7.1 cm [14]. Huang et al. analyzed 43 patients with OMA rupture, of whom 31 had OMA history before spontaneous rupture, and the average maximum diameter before rupture was 6.04 cm [13]. In addition, Dai et al. found the cutoff value of maximum diameter of OMA between the ruptured group and the unruptured group (n = 43 vs. 70) was 9.5 cm, which showed a high specificity of 98.6%, but a low sensitivity of 28.6% [11].

The clinical manifestations were various in patients from short term onset to a very severe and lasting pain depending on the size of rupture and leakage amounts of cyst. If the rupture was small and there was only a little leakage, the patients’ symptoms are less severe and could resolve spontaneously after a few minutes to a few hours without an emergency visit. Even they went to the emergency room, there might be no significant pelvic free fluid accumulation on ultrasound examination.

After the OMA developed spontaneous rupture, the levels of serum HE4 [15], CA125, CA199 and D-dimer will increase, of which the level of CA125 can be as high as thousands [12]. For the diagnosis of spontaneously ruptured OMA in laboratory testing, Dai et al. recommended the combination of CA199 and CA125. Shuang et al. recommended the triple test of CA125, CA19-9 and D-D, in which the double test of CA19-9 and D-D has the highest cost performance [11, 12]. Because of the differences in enrolled participants and testing time, most patients in our cohort did not observe extremely high CA125 and CA19-9 as previously reported (Table 2).

All the included patients within our cohort had undergone elective surgery. 16/33 (48.5%) patients presented to the emergency department with lower acute abdominal pain but did not underwent emergency surgery due to vital signs were stable and the lower abdominal pain was gradually relieving. In patients with a definite history of OMA, there is debate about whether emergency surgery should be performed immediately when sudden abdominal pain is suspected to be caused by spontaneous rupture. Huang et al. recommend emergency surgery within 72 h, which led to higher future fertility compared surgery after 72 h according to their study [13]. However, we did not know the specific latency time of the surgery group in which more than 72 h. In addition, with a mean follow-up time of 54.30 months, the overall recurrence rate of included patients was as high as 39.5%, and patients with recurrent OMA had a further surgical procedure.

Our study showed that spontaneous rupture of OMA was found in 9.0% of women who underwent elective surgery for OMA, suggesting that rupture of OMA is not rare. According to our study, we advocate that if other severe acute conditions such as appendicitis, ectopic pregnancy, torsion of an ovarian cyst, malignant tumor, and hemoperitoneum can be ruled out, conservative treatment can be considered during suspected spontaneous ruptured OMA if the leakage is not much, the patient's vital signs are stable and symptoms are gradually relieved.

This is the first study of spontaneous rupture of OMA through a cohort study of patients with OMA who underwent elective surgery. Previous studies were more based on emergency surgery. There are also some limitations to our study. Firstly, this was a retrospective research, we can’t get the most accurate maximum diameter of OMA before rupture, and there may also be recall bias about onset of AAP; Secondly, all patients were treated by elective surgery and diagnosed as previous spontaneously ruptured OMA, therefore, the patients treated by emergency surgery were not included; Thirdly, because PUMCH is a tertiary referral hospital, and the gynecologist specializes in the treatment of endometriosis, there remains the risk of selective bias of patients, and the incidence may be relatively higher and is not able to be generalized. Furthermore, as concerned of our patients were all nonpregnant, OMA rupture complicating pregnancy is a rarer and more complicated problem [16], which is not within the scope of our study, and more clinical evidence is needed.

In conclusion, our study showed that spontaneous rupture of OMA was found in 9.0% of women who underwent elective surgery for OMA in a tertiary hospital setting of China, suggesting that rupture of OMA is not rare. The prevalence of spontaneous rupture of OMA in our study was higher than that reported in the literatures. For women with a history of OMA, when acute lower abdominal pain attacks, the possibility of rupture of OMA should be considered, especially for patients with larger cysts. Rupture of OMA is not a rare complication, so that a suitable time for surgical intervention for OMA should be considered.

## Data Availability

The article does not involve sequencing data. Demographic information and basic clinical information are already shown in Tables 1 and 2. Other clinical data used in the study are available from the corresponding author upon reasonable request.

## Abbreviations

OMA: Ovarian endometrioma
PUMCH: Peking Union Medical College Hospital
PEM: Peritoneal endometriosis
DE: Deep endometriosis
r-ASRM: American Society for Reproductive Medicine revised staging system
AAP: Acute abdominal pain
BMI: Body mass index
