# Correction: Clinical features of patients with previous spontaneous rupture of ovarian endometrioma operated electively: a case–control study

**DOI:** 10.1186/s12978-024-01939-2

**Published:** 2024-12-19

**Authors:** Zhiyue Gu, Xiaoyan Li, Yi Dai, Jinghua Shi, Yushi Wu, Chenyu Zhang, Qiutong Li, Hailan Yan, Jinhua Leng

**Affiliations:** 1 Department of Obstetrics and Gynecology, Peking Union Medical College Hospital, Chinese Academy of Medical Sciences and Peking Union Medical College, No. 1 Shuaifuyuan, Dongcheng District, Beijing, 100730 China; 2 National Clinical Research Center for Obstetric and Gynecologic Diseases, Beijing, China

**Correction: Reproductive Health (2023) 20:156** 10.1186/s12978-023-01702-z

The authors wish to highlight an amendment to the Results sub-section of the Abstract in which ‘P = 0.003’ should instead state ‘P = 0.007’.

The authors also wish to note that the grant number 86071628 in the original article [1] should instead read as 82071628.

## References

[1] Gu Z, Li X, Dai Y, Shi J, Wu Y, Zhang C, Li Q, Yan H, Leng J. Clinical features of patients with previous spontaneous rupture of ovarian endometrioma operated electively: a case–control study. Reprod Health. 2023;20:156. 10.1186/s12978-023-01702-z. 37865796 10.1186/s12978-023-01702-z PMC10589996

